# Antiplatelet therapy in patients undergoing oral surgery: A systematic review and meta-analysis

**DOI:** 10.4317/medoral.22708

**Published:** 2018-12-24

**Authors:** Julio Villanueva, Josefina Salazar, Ana Alarcón, Ignacio Araya, Nicolás Yanine, Stefan Domancic, Alonso Carrasco-Labra

**Affiliations:** 1 Oral & Maxillofacial Department. Cochrane Associated Center at School of Dentistry. University of Chile; 2 Department of Health Research methodology, Evidence and Impact. Mc Master University, Hamilton, Ontario, Canada

## Abstract

**Background:**

The number of patients under antiplatelet therapy (APT) continues to raise as current recommendations foster this practice. Although some recommendations to manage this treatment during oral surgery procedures exist, these have methodological shortcomings that preclude them from being conclusive.

**Material and Methods:**

A systematic review and meta-analysis of the best current evidence was carried out; The Cochrane Library, EMBASE and MEDLINE databases were searched for Randomized Controlled Trials (RCT) concerning patients undergoing oral surgery with APT, other relevant sources were searched manually.

**Results:**

5 RCTs met the Inclusion criteria. No clear tendency was observed (RR= 0.97 CI 95%: 0,41–2,34; *p*=0,09; I2= 51%), moreover, they weren’t clinically significant.

**Conclusions:**

According to these findings and as bleeding is a manageable complication it seems unreasonable to undermine the APT, putting the patient in danger of a thrombotic event and its high inherent morbidity, which isn’t comparable in severity and manageability to the former.”

** Key words:**Antiplatelet therapy, aspirin, oral surgery, platelet aggregation inhibitors, oral surgical procedures, systematic reviews.

## Introduction

Blood flow obstruction by a clot may cause ischemia and organ infarction. Thrombus formation is produced as consequence of vascular injuries, activation of the clotting process and blood flow disruption, this can happen at venous or arterial level. In arterial thrombosis the main etiologic factors are platelet activation and injuries to the arterial wall such as atheromatous plaques producing platelet rich thrombi. Blood Stasis and clotting are the main factors in venous thrombosis, producing thrombi rich in fibrin and erythrocytes (1).

Atherothrombosis, i.e., thrombus formation over an already present atherosclerotic plaque, causes cardiovascular diseases (2). The most important are stroke, coronary disease and peripheral vascular disease (3). Nowadays, these are the top mortality causes worldwide (4-6). The World Health Organization has declared that in the year 2030 approximately 23.6 million people will die every year due to cardiovascular complications (2,3).

Therefore, antiplatelet therapy (APT) for a number of thrombotic conditions has increased in the last years in primary prevention (prophylactic) and secondary prevention (1).

Currently available antiplatelet agents include acetylsalicylic acid (aspirin), thienopyridines (clopidogrel, ticlopidine) IIb/IIIa platelet receptor inhibitors and phosphodiesterase inhibitors, which act upon the different phases of activation (2). The protective effects of APT against cardiovascular disease have been clearly and concisely demonstrated throughout the groups at higher risk (2).

Treatments carried out in the oral cavity, especially those that may cause blood extravasation, imply a high risk of perioperative bleeding in patients with an altered hemostasis (7,8). Although a 90% of minor postoperative bleedings are due to local factors such as the anatomical situation, excessive surgical trauma and/or not following postoperative indications, most of the severe bleedings are related with systemically alterations that compromise the primary or secondary hemostasis mechanisms (7).

In the light of these issues it must be considered whether if it is necessary to halt or modify the treatment in attention of the higher risk of a non-manageable event of bleeding, peri or postoperatively (9) considering that even a transitory interruption of this kind of medication may lead to a thrombotic event with a worst final outcome (10).

There are a number of published articles in the medical literature intended to underpin the clinical decisions regarding this issue, however, most of them are methodologically flawed, making it hard to properly support such decisions. Therefore, it is necessary to identify, assess and synthetize all the evidence available through a systematic method that could clarify and improve decision making, defining if it is necessary to modify APT in patients undergoing oral surgery in consideration of the known risks and benefits.

With this goal, a systematic review of the literature was carried out. The PICO question was: Patients: Undergoing oral surgery under Antiplatelet Therapy (APT). Intervention: Suspension of APT. Comparison: Maintained APT. Outcome: Risk of perioperative bleeding.

## Material and Methods

A systematic review of the literature and a meta-analysis was conducted. In August of 2017, a structured and systematic search for randomized controlled trials regarding APT and oral surgery was made.

Inclusion: Randomized controlled clinical trials regarding bleeding following oral surgery with suspension or maintenance of anti-platelet therapy.

Exclusion criteria: Non-randomized or non-controlled clinical trials, observational studies, narrative reviews, case reports and letters to the editor, outcome or comparison analyzed didn´t match the inclusion criteria.

The databases searched were THE COCHRANE LIBRARY, MEDLINE and EMBASE (annex 1), Also, a search was carried out in databases of clinical trials such as: Current Controlled Trials (http://www.controlledtrials.com/), ClinicalTrials.gov (http://www.clinicaltrials.gov/), WHO International Clinical Trials Registry Platform (ICTRP) (http://www.who.int/ictrp/en/). The reference list of the articles included was also searched for articles eligible for this review. A search was also conducted in Google Scholar in order to detect grey literature. In journals of the specialty, given the belated indexation of articles in databases, the issues published in the last 6 months were also reviewed, the reviewed journals were: Journal of Oral and Maxillofacial Surgery; International Journal of Oral and Maxillofacial Surgery; British Journal of Oral and Maxillofacial Surgery; Journal of Craniofacial Surgery; Head & Neck: Journal for the Sciences & Specialties of the Head and Neck. Finally, the last 5 years of online summaries from the American Association of Oral and Maxillofacial Surgeons and International Association of Dental Research meetings were manually searched. All the potentially relevant articles found were numbered, afterwards, they were filtered based on their title and summary. This process was performed by 2 reviewers independently, following the inclusion and exclusion criteria previously determined, being these widely inclusive overall.

Subsequently, all the articles selected were submitted to a full-text appraisal. In the same fashion as the previous stage of selection described, 2 independent reviewers carried out the assessment in accordance with the inclusion and exclusion criteria previously determined. The inter-examiner agreement was evaluated with the kappa index (κ= 0,691). Any disagreement was sorted out by consensus or, when it wasn´t possible, by a third reviewer that acted as arbiter.

**Figure 1:**
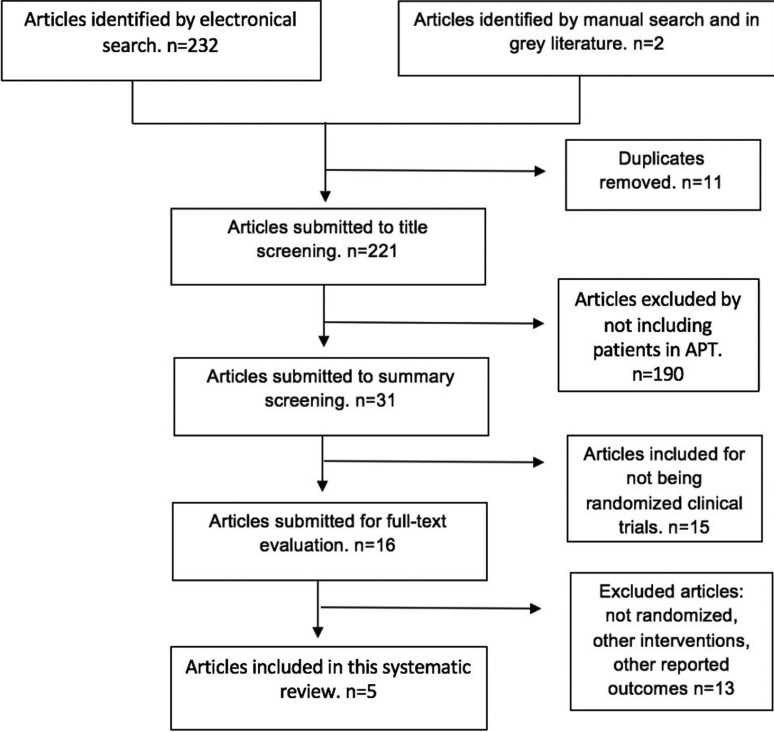
Flowchart depicting study selection.

A flowchart is included (Fig. 1), following the recommendations of the PRISMA Statement (11), in order to illustrate the results of the study selection for this review.

DATA EXTRACTION: In a data extraction form, 2 reviewers independently handled the data aided by a Microsoft ® Excel Office 2016 template designed with this purpose. Disagreements were checked by both reviewers and when consensus couldn´t be reached a third reviewer acted as arbiter. The form registered the following: (i) Information regarding the study characteristics: number of patients included, number of intervention and control groups, intervention of interest, control interventions and counter interventions, (ii) Information regarding the results: bleeding definition, (iii) Bias information: selection bias, performance bias, detection bias, etc.

**Figure 2:**
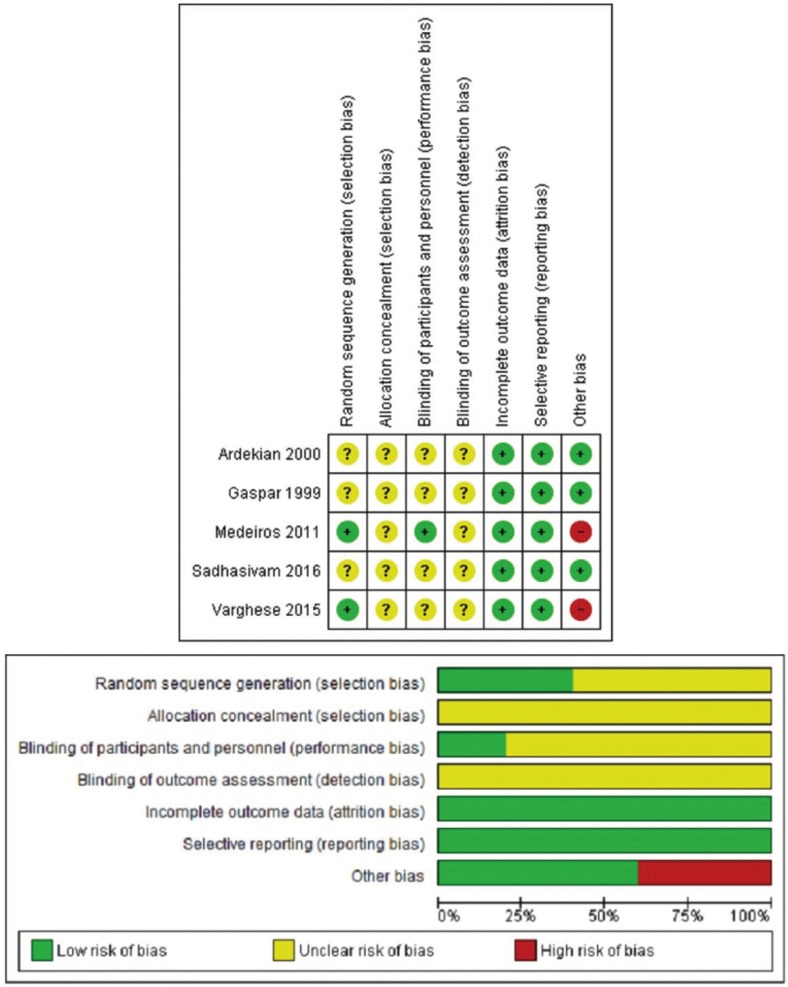
a) Summary of Risk of Bias: Assessment of the reviewers of the Risk of Bias in each domain for each study. b) Risk of Bias Chart: Assessment of the reviewers for each domain of each article, shown as percentages.

RISK OF BIAS ASSESSMENT: The Risk of Bias of the included articles was assessed using the Cochrane Collaboration´s Risk of Bias Tool (12). The assessment was made by 2 reviewers independently reading the full-text of the included articles. Disagreement was discussed and agreement was reached in order to classify the articles in either high, low or unclear risk of bias. It has already been demonstrated that author blinding or study affiliation does not influence results assessment (13), therefore, the reviewers weren´t blinded to this data. Results were presented following the PRISMA recommendations (Figs. 2a,b).

The following effect measurements for the treatment were used to assess the outcome for each study: Bleeding: Relative Risk (RR) was calculated for the effects of treatment (dichotomous outcomes), with a confidence interval of 95% or differences of the risks at their confidence intervals at 95%.

STUDIES´S HETEROGENEITY ASSESSMENT: The chi-square test was used in order to determine the presence of statistical heterogeneity, a 0,1 significance level was used. Quantification of the inconsistency was made by the statistical I2 test, following the Cochrane recommendations (14). Clinical heterogeneity was assessed considering the patient´s characteristics, environment and intervention, consulted with experts. Methodological heterogeneity was assessed by the Risk of Bias Tool´s domains (14).

DATA SYNTHESIS: A narrative description of the included trials´s characteristics is provided. Also, a meta-analysis of the included data results generated by the RevMan 5.1 software is presented. In comparisons were no clinically apparent heterogeneity was found and the I² value is 40% or less, a fixed effects model was used. When clinical heterogeneity was present and the I² value is more than 40%, a random effects model was used (15).

SENSITIVITY ASSESSMENT: Given that allocation concealment and blinding was the most critical bias domain in this review, the trials were classified as at high risk of bias, if the assignment concealment domain and two more domains had high risk of bias. The same criteria was applied to classify an essay as having unclear risk of bias. We included all studies, in spite of the high risk of bias. Because the GRADE tool (16) is used to define the quality of the evidence allowing us to incorporate every study in the review.

QUALITY OF EVIDENCE ASSESSMENT: The quality of evidence for the result (bleeding) was classified using the GRADE Guidelines, accordingly, an evidence profile was included in  Table 1 (16).

**Table 1:**
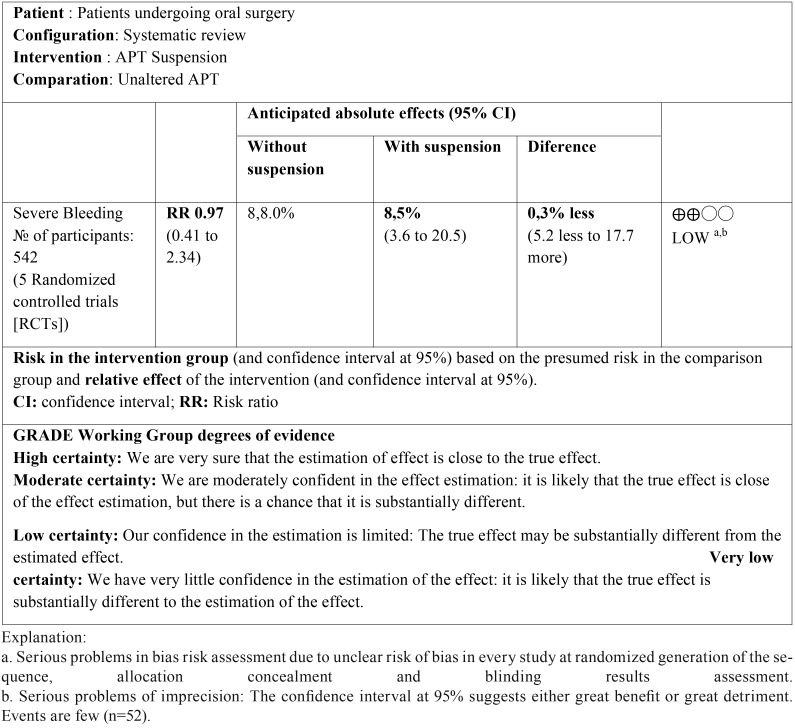
GRADE Evidence Profile for APT suspension compared with unaltered APT in patients undergoing oral surgery.

## Results

The electronic search brought 232 references and the manual search added 2 more published articles. After the exclusion of duplicates 223 articles were submitted for screening of their titles and summaries. 20 articles were deemed potentially relevant. Once the full text was analyzed, a total of 5 articles were included in this review: Gaspar, 1999 (17); Ardekian, 2000 (18); Medeiros, 2011 (19); Varghese 2015 (20) and Sadhasivam 2016 (21) ( Table 2,  Table 2 continue: Characteristics of included studies). Of the 20 articles in which its full text was reviewed, 15 were excluded from this review as they didn´t meet the inclusion criteria ( Table 3,  Table 3 continue). From the included studies, 2 were conducted in Israel (17,18), 1 in Brazil (19), 2 in India (20,21). Only one specifies in which setting it was conducted, being that, a medical center (18).

**Table 2:**
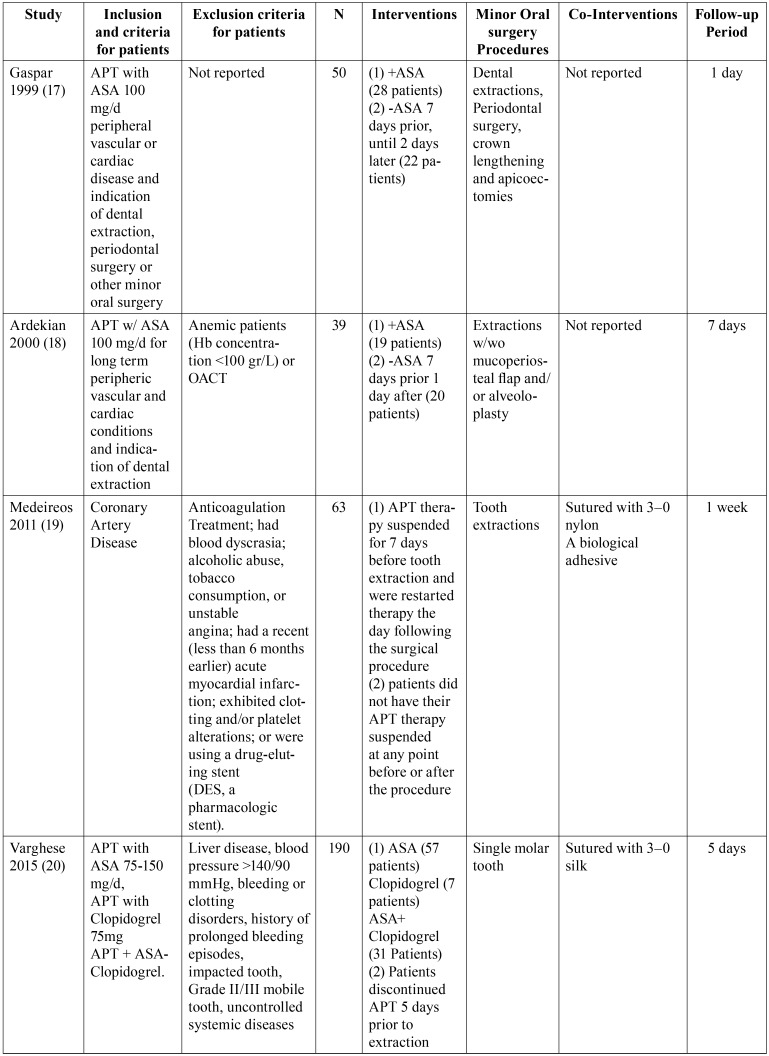
Characteristics of the Included Studies. APT= Anti-Platelet Therapy; ASA= Acetyl Salicylic Acid; N= Number of patients; (+) = Continues APT; (-) =Suspended APT; OACT= Oral Anti-Coagulation Treatment.

**Table 2 continue:**
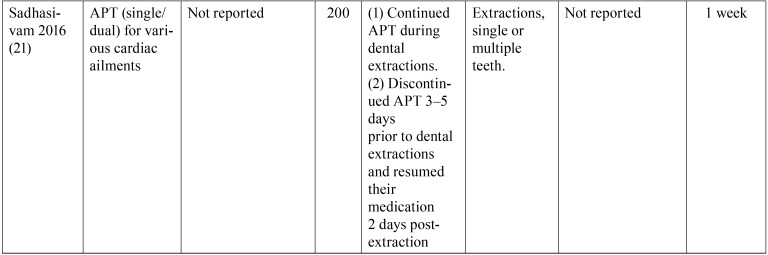
Characteristics of the Included Studies. APT= Anti-Platelet Therapy; ASA= Acetyl Salicylic Acid; N= Number of patients; (+) = Continues APT; (-) =Suspended APT; OACT= Oral Anti-Coagulation Treatment.

**Table 3:**
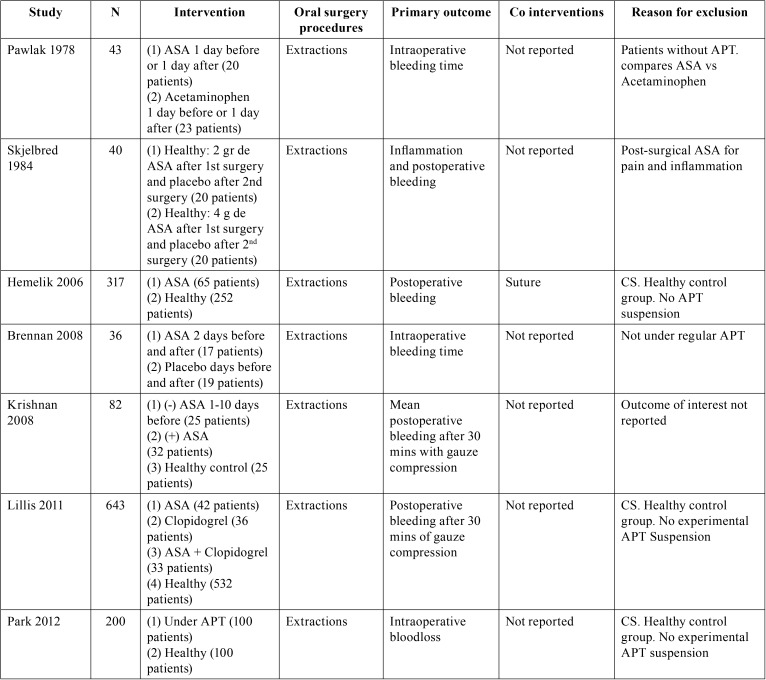
Characteristics of excluded studies and reasons for exclusion, following full-text screening. APT= Antiplatelet Therapy; ASA= Acetylsalicylic Acid; N= number of patients; (+)= continues APT; (-)= APT suspension. CS= Cohort Study.

**Table 3 continue:**
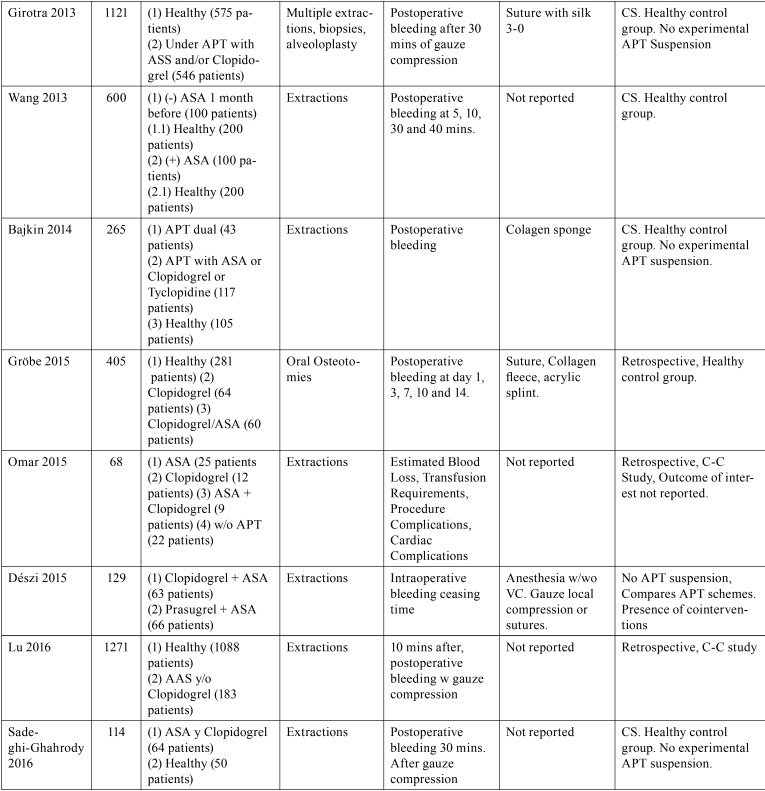
Characteristics of excluded studies and reasons for exclusion, following full-text screening. APT= Antiplatelet Therapy; ASA= Acetylsalicylic Acid; N= number of patients; (+)= continues APT; (-)= APT suspension. CS= Cohort Study.

The full number of patients included in the studies was 542, this ranged from 39 (18) to 200 (21). Demographical data was reported in the 3 studies, male participants were in higher proportion than females and the mean age of the participants was between 58 years (19) and 64 years (18).

The type and number of oral surgery procedures was variable among the studies, among these were included, dental extractions only (19-21), single or multiple. The other studies comprised single tooth extractions with or without alveoloplasty (18) as well as other minor oral surgery procedures such as apicoectomies and periodontal surgery (18).

The inclusion criteria for participants were reported in three studies (17-19) and was not informed in two studies (20,21). All of them included patients in APT with acetylsalicylic acid. Exclusion criteria was not informed in two of these (18,21). The exclusion criteria in the other three studies are concomitant treatment with oral anticoagulation drugs, alcoholism, liver disease, impacted tooth and blood dyscrasias (18-20).

All the trials had parallel groups and the effects of maintaining the APT in contrast with suspension of the former 7 days and 3-5 days prior to the procedure (17-21) 

Five of the included studies evaluated intense bleeding events. All had the patient monitored and evaluated by the treating professional during the first 24 hours following the procedure. Three studies reported a follow-up period of 7 days (17-21).

There is no universally accepted standardized definition of bleeding in patients undergoing oral surgery, therefore the outcome of interest was described by 3 of the studies as intraoperative bleeding, being determined by subtraction of the irrigation fluid from the blood accumulated in the suction flask (18,19,21). The result in these studies was reported as intense bleeding.

No study reported the occurrence of stroke, an ischemic cardiac event, nor quality of life assessment following the intervention.

RISK OF BIAS: The risk of bias assessment is displayed in Figure 2. No study was classified in every domain as low risk of bias.

The domain considered as at low risk of bias was the selective report of results, and the incomplete report of data (Fig. 2).

Only two studies gave details regarding the randomized sequence generation; therefore, the other three were classified as studies with unclear risk of bias in this domain.

No study reported information regarding the allocation concealment, therefore they were classified as studies with an unclear risk of bias in this domain.

This domain was evaluated in 2 areas, blinding of the patients and personnel (performance bias) and blinding of the outcome assessors (detection bias). Regarding performance bias, only 1 study describes blinding (19). No study gave information about the assessor´s blinding, therefore they were classified as unclear risk of bias in this domain.

Five studies were classified as low risk of bias, as the criteria for intraoperative bleeding assessment (17-21).

Two studies had co-interventions, considered as a high risk in other sources of bias. These were use of suture, biological adhesive and local anesthetic 3% percent.

Outcome: Perioperative bleeding

The global estimator for intense bleeding was reported for patients undergoing oral surgery in a group that suspended the APT in contrast with a group in which no suspension was indicated, the findings are in terms of relative risk (RR=0,97); with a 95% confidence interval (CI), ranging from 0,41 to 2.34. Heterogeneity: Chi square test= 8.16; *p*=0,09 and the Inconsistence test I2=51%. As there was apparent clinical heterogeneity, having an I² value higher than 51%, a random effect model was applied.

**Figure 3:**
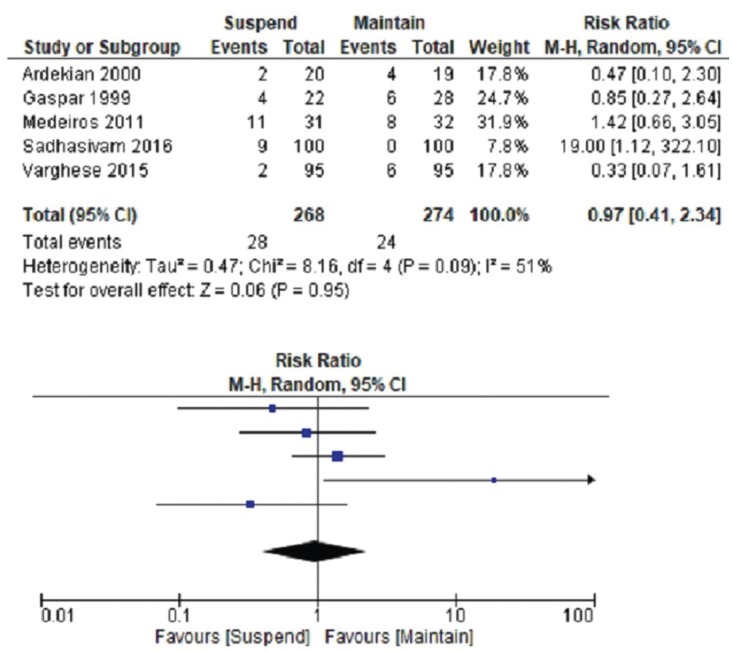
Forest Plot: Each square represents a punctual estimator (Relative Risk) for each study, while the diamond at its mid-section, represents the Global Estimator (0,97). The horizontal lines that cross the boxes illustrate the magnitude of the confidence interval at 95%. The diamond width serves the same purpose (0,41-2,34). The vertical line depicts the line of no-effect, depicting a neutral RR. The horizontal spacing between a box and the vertical line shows the difference between the experimental group and the control in relationship to a neutral effect. The size of each square depicts the weight of each study, indicating the relevance of each one in the global estimation.

META-ANALYSIS: The meta-analysis is shown in Figure 3.

EVIDENCE QUALITY ASSESSMENT: The protective effect of APT suspension over perioperative bleeding in patients undergoing minor oral surgery procedures was qualified as low-quality evidence according to the GRADE domains ( Table 1).

## Discussion

The fear of a postoperative hemorrhagic event is well known in medical and dental practice. Fortunately, minor oral surgery jeopardizes anatomical sites that favor hemostatic control. These sites are frequently surrounded by bone tissue, meaning that there more possible areas over which pressure can be applied as well as other hemostatic measures, also, there is usually less soft tissue that could generate bleeding, although irrigation is vast in this area. Moreover, the oral cavity is easy to supervise for the patient as well as the clinician, which enables early detection and opportune treatment of any possible complication (22).

Likewise, diverse studies regard postoperative bleeding cases mostly manageable with routinary hemostatic measures, such as pressure with a gauze, hemostatic material application, sutures with or without pro hemostatic agents like tranexamic acid (40); Namely, it has been reported that the incidence postoperative bleeding that cannot be managed by these measures varies from 0 to 3,5% (23,24).

In the last decades, APT prevalence has grown considerably and is ever more frequent given the increased number of patients at risk of developing pathological entity related to a thrombotic event, as well as the number of cardiac surgeries involving prosthetic valves or coronary stents (25,26).

The main risk of APT in the patient to be submitted to a surgical procedure is the risk of developing an uncontrollable hemorrhagic event.

It is essential to recommend and take clinical decisions before a patient in APT, to consider the consequences of possible hemorrhagic events during surgery bearing in mind the possible consequences of discontinuing the APT. Kearon and Hirsh observed in a comparative study approximately 20% of the cases of arterial thromboembolism are fatal and a 40% result in permanent disability (27). Another study reports that premature dual APT interruption is the leading cause of late thrombosis in patients submitted to angioplasty and stent installment (28). Furthermore, there are over 50.000 of patients where aspirin cessation is associated with a threefold risk of developing cardiac events (29).

In this scenario, considering the toll that thrombotic events carry over a patient´s health, it would be reasonable to think of performing a surgical intervention without alteration nor suspension of the antiplatelet therapy, provided that it wouldn´t represent a significant risk of hemorrhagic complications. The purpose of this review is to determine if the APT is to be suspended, once thrombotic and hemorrhagic events are considered.

In this systematic review it is evident, regarding the risk of bias, that none of the studies included described in a sufficiently clear manner the domains presented in the assessment tool, as to be classified as a study with a low risk of bias. On the other hand, after carrying out the meta-analysis, it could be seen that the included studies elicit a heterogeneity level of I2=51% with a non-significant p value (p=0,09), however, as not every aspect needed to protect the methodological quality of the clinical trials is explicated, it is not possible to presume that such methods were in fact employed in order to control the risk of bias, which compromises the precision of the results. This is why a random effect model was used in this case.

Individual results of each study are not sufficiently strong such as to favor APT suspension before oral procedures, because the confidence intervals of the included studies are too wide, showing unprecise results to the point in which all of them cross the no-effect line. In addition, the risk of bias is high among the studies as a whole, ergo, these results are not clinically nor statistically significant.

Regarding the global estimator obtained from the meta-analysis, it is observed that it does not favor suspension or maintenance of the APT in order to avoid intense intraoperative bleeding furthermore, the resultant confidence interval crosses the neutral result line (RR=1), hence it is not clinically significant.

A point worthy of discussion is the definition of a bleeding event. There is no consensus to define and measure bleeding after an oral surgery procedure in patient under APT (30). Lockhart (31), suggested that an event of bleeding is considered significant if it meets 1 of 4 criteria: bleeding that continues after 12 hours, that forces the patient to call or to head back to the office or an urgency service, that results in the development of a considerable hematoma or ecchymosis in the oral soft tissues or, that requires blood transfusion (31). None of the studies included in this review reported such an event.

For this systematic review the information available in the literature regarding invasive oral procedures conducted in patients under APT was appraised. And only 5 studies were found eligible in accordance with the inclusion criteria, nonetheless, there is a vast number of observational studies that in spite of not answering to the question posed in this review, render relevant knowledge to the topic. The scarcity of randomized clinical trials may well be attributed to the ethical dilemma generated by the possibility that patients, that have no indication to be enrolled in a trial, could be assigned to an intervention that puts them at risk of major adverse outcomes.

It is important to note that only one of the included studies in this review considered patients under dual APT (21), therefore the results mainly portray the effects of ASA suspension. It is possible to conclude for these patients that the currently available evidence is not sufficient to settle that the suspension of the APT leads to a beneficial effect over hemorrhagic events prevention. Given the available methods at disposal for perioperative bleeding control, it would be advisable to keep the APT unaltered in attention to the associated risks of its interruption and foreseeable consequences.

There is even less existent evidence relative risk of bleeding with other drug than aspirin or dual APT (32,33). Dézsi (10), described an increased bleeding time in patients under dual APT with clopidogrel or prasugrel, in contrast with the patients under aspirin monotherapy (10). What is more, it has been reported that patients with combined anticlotting drugs and aspirin elicit an increased risk of suffering postoperative complications (33). Accordingly, the management of this patients requires additional considerations.

The present systematic review exhibits limitations regarding the number and quality of the included studies. Studies with a bigger sample size and better design are necessary in the future. This encompasses, better methodological quality in order to reduce the risk of bias, elimination of confounding variables and a clear definition of the parameters required to evaluate hemorrhagic events, so as to obtain results with broad clinical applications, allowing the evidence to effectively underpin clinical practice.
